# Syndemic contexts: findings from a review of research on non-communicable diseases and interviews with experts

**DOI:** 10.1080/16549716.2021.1927332

**Published:** 2021-07-25

**Authors:** Irene Pirrone, Marjolein Dieleman, Ria Reis, Christopher Pell

**Affiliations:** a Faculty of Science, Vrije Universiteit Amsterdam, Amsterdam, The Netherlands; b Faculty of Science, Athena Institute, Vrije Universiteit Amsterdam, Amsterdam, The Netherlands; c Department of Public Health and Primary Care, Leiden University Medical Centre, Leiden, The Netherlands; d Department of Anthropology, University of Amsterdam, Amsterdam, The Netherlands; e The Children’s Institute, University of Cape Town, Cape Town, South Africa; f Amsterdam Institute for Global Health and Development, Amsterdam, The Netherlands

**Keywords:** Syndemics, context, conceptualisation, methodology, interdisciplinarity

## Abstract

**Background:**

Syndemics are characterized by the clustering of two or more health conditions, their adverse interaction, and contextual factors that create the conditions for clustering and/or interaction that worsens health outcomes. Studying syndemics entails drawing on diverse disciplines, including epidemiology and anthropology. This often means collaboration between researchers with different scholarly backgrounds, who share and – ideally – integrate their findings.

**Objective:**

This article examines how context within syndemics has been defined and studied.

**Methods:**

A literature review of empirical studies focusing on syndemics involving non-communicable diseases (NCDs) and mental health conditions was conducted and the full text of 13 articles was analyzed. The review was followed-up with semi-structured interviews with 11 expert researchers working in the field.

**Results:**

The review and interviews highlighted a relatively consistent definition of syndemics. The reviewed studies of NCD-related syndemics tended to focus on micro-level context, suggesting a need to analyze further underlying structural factors. In their syndemics research, respondents described working with other disciplines and, although there were some challenges, welcomed greater disciplinary diversity. Methodological gaps, including a lack of mixed methods and longitudinal studies, were identified, for which further interdisciplinary collaborations would be beneficial.

**Conclusions:**

NCD-related syndemics research would benefit from further analysis of structural factors and the interconnections between syndemic components across multiple levels, together with more ambitious research designs integrating quantitative and qualitative methods. Research on the COVID-19 pandemic can benefit from a syndemics approach, particularly to understand vulnerability and the unequal impacts of this public health crisis.

## Background

The medical anthropologist Merrill Singer first introduced the term syndemic in the 1990s to define the interactions between substance abuse, violence, and AIDS (SAVA) [1–3]. Singer first referred to the SAVA syndemic as ‘a closely interrelated complex of health and social crises’ [1]. Later, he identified three necessary criteria to characterize a syndemic [2,4]: the clustering of two or more diseases or health conditions, such as infections, mental health problems, and/or non-communicable diseases; biological interactions among the health conditions that lead to an increased health burden in the affected population; and contextual factors that create the conditions for clustering and/or for interaction that worsen health outcomes.

The focus on the complex interplay among biological and contextual factors characterizes the syndemic approach [5]; adverse contextual factors, such as poverty, violence, limited access to care or stigma, play a central role in facilitating the clustering of some health conditions and worsening their pathogenesis and treatment efficacies [5–7]. For example, financial insecurity in low-income populations contributes to stress and depression, which biologically interact with diabetes pathogenesis, and has been associated with poor adherence to treatment in diabetes patients [7]. Furthermore, risk factors for both conditions often cluster in contexts of social and economic marginalization thereby facilitating diseases interactions [7].

Studying syndemics is challenging because it requires research strategies that address biological *and* social processes [8]. Syndemic research also calls for multi-level analyses to understand interactions at the individual and population level [9]. Hence, comprehensively examining syndemics as inextricably entangled complexes of phenomena benefits from integrating methodologies and knowledge from diverse fields [4,7,10]. Achieving such interdisciplinary often means researchers with different backgrounds sharing and – ideally – integrating their approaches and findings [11]. To do so, they need to find ‘common ground’ in assumptions and concepts that are relevant for developing new knowledge [11]. In syndemics research, key aspects, such a context, likely attract contrasting conceptualizations and operationalizations across disciplines [4,12]. However, little is known about whether and how syndemics researchers from different disciplines have managed these tensions.

Originally focused on vulnerable communities in high-income countries (HICs) [1], more recently, a syndemics approach has been applied in low- and middle-income countries (LMICs) [13–15]. Here, epidemiological transitions mean that non-communicable diseases (NCDs) are co-emerging and interacting with other health conditions, such as infectious diseases, in settings shaped by instability [7,15]. Researchers, such as Mendenhall et al. [7,16], are bringing attention to NCDs and mental health conditions in countries where policies and research often prioritize infectious diseases or disease-specific interventions [17].

Drawing on a literature review of syndemic research involving mental health conditions and NCDs, and interviews with experts in the field, this article examines how context has been conceptualized and applied in syndemic research. Syndemics involving NCDs and mental health conditions were selected because this is a relatively under-studied area (compared to infectious disease-related syndemics) yet these conditions are responsible for a significant burden of global morbidity and mortality: 71% of all deaths per year according to the World Health Organization [18]. The article focuses on context because it is a slippery concept, which is understood and addressed in diverse ways across disciplines engaged in syndemics research [4,7].

## Methods

Data were collected as part of a systematic literature review and interviews with experts in the field. The literature review focused on syndemics involving mental health conditions and NCDs, due to the recent growth of research in this area and its relevance for LMICs [16]. They provide a case-study to explore the state of the art of the study of context in syndemics research. Subsequently, interviews with experienced researchers were conducted to complement the literature review findings and to gain more insights into researchers’ perspectives on interdisciplinarity and its challenges within syndemics research.

### Literature review

#### Search strategy

On 15 April 2020, the literature search was conducted in PubMed, AnthroSource, CINHAL, and Scopus databases using the search terms: syndemic* AND (NCD OR NCDs OR non-communicable OR ‘metabolic syndrome’ OR diabetes OR diabet* OR ‘chronic respiratory’ OR cardiovascular OR chronic) AND (mental OR depression OR depressive OR depre* OR anxiety OR stress OR psychological). The terms were searched in all fields/text, except for the Scopus search that was conducted in ‘Article title, Abstract, Keywords’ because the number of outputs was more comparable to the other databases outputs and more feasible for this study’s purpose. The search was limited to peer-reviewed articles as document type and English as language. No time frame was set for inclusion because the search generated a manageable number of publications to be screened and they were almost all recent (i.e. within the last 5 years). Further inclusion criteria were: articles based on empirical studies (i.e. include data collection), applying the syndemic concept, and including NCDs and mental health conditions as syndemic components.

#### Data processing and analysis

**Figure 1.**
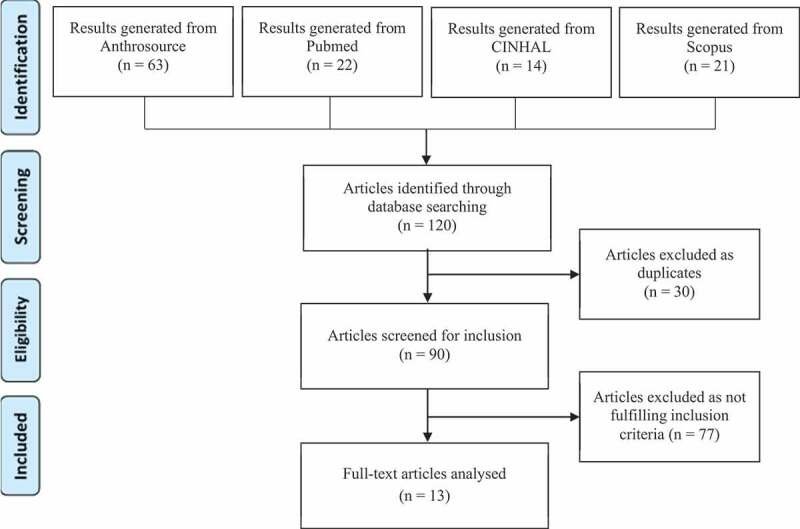
Literature review flow chart

**Figure 2.**
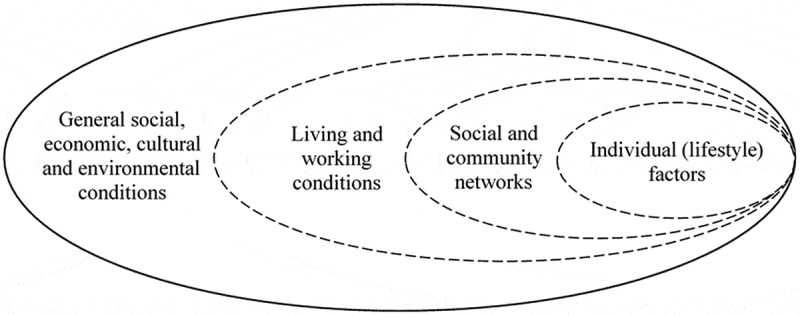
Four layers of context, adapted from the main determinants of health model [19]

As shown in Figure 1, Anthrosource database generated 63 results, Pubmed 22, CINHAL 14, and Scopus 21. All citations were imported into the reference manager program Mendeley. Among the 120 results, 30 articles were removed as duplicates. The title and abstract of the remaining 90 articles were screened for relevance, according to inclusion criteria. The full text of some articles was assessed because the abstract provided insufficient information as to whether NCDs and mental health conditions were addressed as part of a syndemic. Seventy-seven articles were excluded through this selection process. For the selected 13 articles, information was extracted regarding discipline(s) involved, definition and operationalization of key concepts related to syndemic (i.e. syndemic definition and bio-social interactions), level of analysis, factors included in the study of context and the methodology applied. Context conceptualization was structure according to the four layers shown in Figure 2, adapted from the ‘rainbow’ model of the main determinants of health [19]. Contextual factors included in the articles reviewed were linked to the model layers. Additional information on challenges or limitations of the analysis was also extracted.

### Interviews

Interviews with experienced researchers were conducted to complement the literature review findings on context conceptualization and methodologies applied to study context within syndemics. They also addressed the challenges and possibilities experienced by researchers when working on syndemics, particularly related to the study of context using an interdisciplinary approach.

#### Participants

A purposive sampling approach was used to include researchers from varied fields and disciplines working on syndemics. Selection criteria were: (1) participant has published work on syndemics: (2) has worked in a research institute or university; (3) has a PhD degree or is a PhD student. Participants were mainly identified from the literature on syndemics. Additionally, one participant was identified through professional contacts. Snowball sampling was also used: contacted participants were asked for recommendations of researchers that met the inclusion criteria.

#### Data collection and analysis

Semi-structured interviews were conducted to gain insights into researchers’ experiences and opinions of the study of context and interdisciplinarity within syndemics research. Participants were able to talk freely about the topics, express their perspectives and elaborate on specific or new aspects considered relevant [20,p.385]. An interview guide was designed to follow-up on the findings from the literature review, with the main topics included: conceptualization and operationalization of syndemics and context, methodology to collect and analyze data on context within syndemics, challenges in conducting research and interdisciplinarity. Additionally, interviews allowed for a discussion on future improvements for syndemic research and the relevance of the theory to the current COVID-19 pandemic. Due to practical barriers and the COVID-19 outbreak, all interviews were conducted online. Interviews were conducted in English and lasted about 30–50 minutes. Upon agreement, interviews were recorded using audio recording programs. Afterward, interviews were transcribed. Anonymity was assured by mutating all phrases or details that could trace back to participants. The anonymized transcripts were coded in Atlas.ti 8.4.16. Thematic analysis was conducted, with main themes pre-determined (derived from the interview guide topics) and adjustments to the codebook made according to emerging (sub)themes. Afterward, patterns across themes and interviews were identified and analyzed.

#### Ethics and Data Management

An ethical review self-check provided by the Faculty of Science – Vrije Universiteit (VU) Amsterdam determined that no further ethical approval was required for this study. Collected data are considered as low risk, not sensitive and related to researchers’ professional views. Before each interview, an information sheet and consent form were emailed to participants to be signed. If the participant had not signed and returned the form digitally, consent was asked and recorded at the beginning of the interview. All data were collected confidentially and stored in an encrypted and password-protected folder. Key-file with personal information of the participants (name and e-mail address) and consent forms were stored in a separate secured folder. Data were only accessible to the research team.

## Results

Thirteen sources were selected for full-text review [15,16,21–31]. The contextual factors were categorized using the four-layer model (Figure 2), adapted from the ‘rainbow’ model [19]. All but one article included ‘living and working conditions’-related factors. ‘Social and community networks’ were also often included in the analyses. Three articles referred to ‘general social, economic, cultural and environmental conditions’, and five included ‘individual lifestyle factors’. Three studies applied quantitative methods, four qualitative, and six used mixed methods. Further details about the reviewed articles can be found in Table A1 in Appendix A.

Semi-structured interviews were conducted with 11 researchers working on syndemics. Table A2 in Appendix A shows participants’ main characteristics. Participants were seven Americans, two Europeans, one African and one Australian. Most had conducted work in more than one continent. Eight were anthropologists, of whom three described mixed anthropology-public health backgrounds. The other three participants’ professional backgrounds were medicine, psychiatry, and public health nursing. Most respondents had conducted fieldwork; two were also involved in intervention research; and four had been closely involved in the academic debate around theories of syndemics. Respondents could thus focus on different aspects of studying context in syndemics (e.g. practical challenges, debates about theory development).

The following sections include a qualitative synthesis of findings. The findings from the review aligned well with those from the interviews and, therefore, they are presented together and organized thematically.

### Conceptualizing syndemics

Almost all reviewed articles defined a syndemic as characterized by interactions among biological, social and structural factors that negatively affect health and well-being, often occurring in disadvantaged population groups. When defining the concept, they usually cited one of Singer’s works [8,32]. One article [30] defined a syndemic by focusing especially on the adverse contextual factors that facilitate the emergence and exacerbation of multiple health conditions. Only two articles provided no explicit definition of a syndemic [26,29], but the authors’ rationale and citations made it clear that they were well aware of the concept and its meaning. Some articles provided a ‘working’ definition of the specific syndemic under study.

In the interviews, all participants also provided a definition, more or less elaborate, which could be traced back to Singer’s work. One medical anthropologist provided a comprehensive definition:

Syndemics is basically led by three rules: number one, there’s clustering of two or more diseases within a population; number two that there’s interaction at the biological, psychological or social level; and then the third is that it’s driven by some sort of social, economic, political, ecological factors that drives the conditions to clustering and interact (R5. Medical anthropologist)

All participants mentioned the co-occurring and clustering of diseases or epidemics. However, one medical anthropologist specified that it is not always diseases or illnesses that are involved, and therefore it is more appropriate to talk about ‘*biological factors or conditions’*. All participants referred to ‘*social conditions*’ or other context-related terms. Most described the complex interplay among the different components as a defining attribute of a syndemic. Four reported coming across the syndemic concept when trying to make sense of findings that were so intertwined and ‘*spiralling*’ that they were almost impossible to disentangle.

A lot of things were really interconnected so you couldn’t separate one thing from the other. So to me that really spoke to having a syndemic because everything was so interconnected and went around in circles (R3. Medical anthropologist & public health specialist)

Two participants pointed out how the definition has changed over the years. One medical anthropologist, closely involved in syndemic theory development, said that part of the confusion surrounding the use of the syndemic framework could result from the fact that researchers refer to conceptualizations from Singer’s earliest work. This anthropologist highlighted the relevance of recognizing the developments that have been made throughout the years to articulate the concept fully and appropriately. Other participants acknowledged the fact that the term is not always used accurately in the literature and the need to be more precise.

Beyond the syndemic definition, the articles did not provide an explicit and detailed definition of ‘syndemic interactions. Factors were mostly described as ‘*intertwined*’ or interacting through ‘*synergies*’. Mendenhall [15,p.302] explained that synergy means that the interaction ‘*escalates the burden of suffering and disease in a way that exceeds the impact of any single factor’*. Some authors suggested mechanisms for interactions among diseases and social factors, such as ‘*multiplicative interactions*’ [27], ‘cumulative effect’ [25], and ‘bidirectionality’ [23]. Other authors described the bio-social interactions more generally as the ways in which the context facilitates the emergence of health conditions, their clustering and progression [21,22]. Only one article [21] presented a comprehensive and elaborated model with five major mechanisms involved in syndemics to explain the interactions and clustering concepts.

In the interviews, all participants distinguished between interactions among health conditions at the biological level (bio-bio) and interactions between the health conditions and the social, economic or political conditions (bio-social). Not all participants were clear about the influence of contextual factors on the biological conditions, linking it back to the complexity of interconnections among syndemic components. A few pointed out that it is crucially important to focus on ‘*how structural factors impact biological-biological interactions’*, articulating how these factors contribute to the clustering and/or interaction of biological conditions. One epidemiologist/medical anthropologist described how there is an open debate in the field regarding ‘interactions’, which might also favour multiple operationalizations of the concept.

### Context: concept and operationalization

Most participants offered a definition of context that resembled the social determinants of health model, that is, including all the social, cultural or economic conditions that shape a population’s environment and, in one way or another, influence people’s health and well-being. Some differentiated individual or micro-level context (living conditions, housing and daily surroundings) from macro-level (e.g. political or societal characteristics). Two participants (anthropologists) defined context as ‘*fluid*’, with one stating that there is no single definition, but rather, ‘*it’s really up to any researcher to define what they mean by context’*. Other participants agreed by saying that researchers have to define the context of their study looking at the specific factors that affect that particular population under study.

History matters, culture matters, politics matters, environment matters, society matters, what policies are implemented matters, but what matters for what is not always the same (R5. Medical anthropologist)

When asked if they could identify the most important contextual factors in syndemics, all participants agreed that prioritizing some factors is not appropriate and that researchers should try to understand them all together, and the interplay of different levels. Given that context can be very broad, in general, participants highlighted the importance of looking at the factors that specifically distinguish the population group under study from others. Different participants considered these factors a good starting point for understanding context within a syndemic and for trying to intervene. Almost all participants referred to contextual factors that affect marginalized or vulnerable populations, such as different kinds of inequality, violence, discrimination and exclusion. Poverty or economic stress was also often mentioned.

Almost all reviewed articles included contextual factors that can be linked to ‘*living and working conditions*’ and ‘*social and community networks*’ layers [19]. Income, employment status and education were the most common factors included. Some articles elaborated on living and working environments (e.g. safety, noise, or pollution). Relationships with family members, often shaped by violence and abuse, were another common aspect. Three articles included ‘general social, economic, cultural and environmental conditions’ in their analysis. For example, Bosire et al. [29] explicitly included access to care and international donor policies in the analysis. A small number of articles included ‘individual lifestyle factors’, such as tobacco use and food-related choices. Some recognized – more or less explicitly – that these factors need to be investigated by focusing on the circumstances, often shaped by poverty and marginalization, that may influence them, rather than being treated as personal choices.

Participants’ research on context within syndemics reflects this description: most had focused on micro-level contextual factors, which commonly included living conditions (e.g. housing and neighborhood); socio-economic indicators, such as income, education and (un)employment; family and community environment; and access to resources (e.g. food, health care). A few participants included broader political, economic, policy or cultural identity-related aspects that characterized the population under study. Two participants (epidemiologist/medical anthropologist, and public health anthropologist) said that they would first look at structural level factors, such as policies or laws, and structural changes and then look at the individual-level factors and outcomes. Interviewees described how micro-level factors are ‘produced’ by the broader socio, economic, political context. They highlighted how multiple pathways and interconnections are often involved, and the difficulties of disentangling these relationships.

Although most of the participants recognized the importance of including macro-level factors, they also admitted the difficulties in doing that, both in research and interventions.

I think at the end of the day that’s a huge driver of the context [the broader context like politics and economic conditions, ed.] that we as researchers have a hard time studying because they’re hard to manipulate (R9. Public health nurse)

This may also explain the trend to focus primarily on micro-level contextual factors, encountered in the reviewed literature, and recognized by different participants. One anthropologist particularly highlighted a tendency among researchers working in public health to neglect the higher level ‘structural circumstances’ involved in syndemics.

Five participants recognized that syndemic research has often focused primarily on micro-level contextual factors and the need to include and analyze broader contextual factors, with one mentioning the importance incorporating the ‘natural environment’ and other describing how culture was often ‘understudied’. One participant (medical anthropologist) specified that, instead of referring to ‘social or psychosocial factors’ within syndemics, the more appropriate term would be ‘*structural factors*’, that is, the underlying macro-level factors. This would indicate that what matters to the theory is being able to explain what is producing the adverse ‘micro-level factors’ in the broader social, economic or political context. History and how contextual factors have changed through the years were described as relevant for further study. Some also mentioned that protective factors and environmental aspects, for example, climate change and air pollution, would be interesting to include more to have a broader perspective on context.

### Methods

Because when we’re studying things that are so complex such as syndemics, that have so many multiple factors influencing, I really do think that it leads ourselves to mixed methods kinds of approaches to combine different sources (R9. Public health nurse)

Most participants agreed that qualitative and quantitative methods are relevant to study context within syndemics. Given the complexity of syndemics and the different levels of context that can be studied, participants specified that using multiple methods is necessary to obtain a comprehensive understanding. At the same time, most participants described qualitative methods as necessary for an in-depth understanding of the factors involved.

Most reviewed articles drew on mixed- or qualitative methods. For both study designs, interviews were the main method used to investigate life-history narratives. This reflected the experiences of participants. Through an in-depth understanding of the lived experiences, these methods helped to identify the main (contextual) factors involved in the specific setting and their interconnections. One medical anthropologist added that ethnographic work needs to be scaled up and complemented with epidemiological and population-level analysis.

In addition to interviews, mixed-methods studies from the review included anthropometric measurements (height and weight), blood test analysis (usually HbA1c for diabetes), and questionnaire/surveys (mostly to assess depression symptoms, anxiety, or diabetes, and to collect sociodemographic information. Page-Reeves et al. [25] also used structured-dialog group sessions as an additional qualitative method to allow participants to elaborate on the main themes under analysis. Most participants involved in fieldwork reported using questionnaires, including depression or disability scales, and anthropometrics.

Information on data analysis was more difficult to retrieve from the reviewed articles. All qualitative and mixed-method studies used content analysis to identify main and recurrent themes in interview transcripts. Among the emerging themes and topics, some articles focused on a limited number of them in line with their main research question, to analyze only a part of contextual factors involved in the syndemic under study. For example, Mendenhall [15] focused on structural and interpersonal violence among South African black women, whereas Bosire et al. [29] focused on the complexities related to access to care from a hospital in Kenya. In quantitative and mixed-method studies, to examine and measure associations among contextual factors and health conditions, regression analysis was the main method used. However, the authors applied different models (i.e. generalized linear model, multinomial logistic regression, linear regression).

Analyzing data to capture the interactions among syndemic components, especially between contextual and biological factors, emerged as a complicated issue in all interviews. Most participants had conducted qualitative analysis and described it as digging into the data and the circumstances to try to understand the factors involved, why and how some interactions happen, and to explain the pathways and interconnections. In terms of ‘quantitative’ analysis, participants described an ongoing debate around the most appropriate statistical methods and no specific method emerged as the most appropriate. Almost all participants said that there can be more options, depending on the type of study and the variables, each with its strengths and limitations. A few reported witnessing a trend, especially in epidemiology and public health, to frame the theory around the interaction aspect in a more statistical way. Participants seemed concerned about focusing too much on this ‘quantitative’ debate and on demonstrating a relationship and its consequences.

Different participants acknowledged that interaction is often perceived as a ‘quantitative’ term and this may contribute to confusion. However, in general, participants said that to be more precise it is not necessary to find the correct and best way to prove interactions in a quantitative way, but rather to acknowledge and distinguish the different types of interactions on which one study may focus and/or deepen the level of analysis. As already pointed out, what matters for syndemics, is identifying the structural factors that are at the root of the syndemic circumstances and the way they impact on people's circumstances to understand how and why some interactions occur. Almost all the participants recognized the challenge of analyzing root factors and trying to capture the complexity of their interactions.

Participants agreed that longitudinal studies are needed. More population-level analysis and data were also described as beneficial. Similarly, all reviewed articles were based on cross-sectional studies, although some authors acknowledged that this approach hinders the analyses of complex interrelationships. For example, Diderichsen and Andersen [21] pointed out that cross-sectional analysis cannot be used to draw causal inferences.

### Interdisciplinarity in syndemic research

The relevance of interdisciplinarity in syndemics research was discussed in all the interviews. Participants agreed that, because syndemics involve different components, their study and understanding require expertise in different areas.

Syndemics is a recipe, like identify your structural factor, what are the two biological factors, how they mix together … you have to know what all your ingredients are to be able to make the cake and if one of the ingredients is missing, the cake is not going to turn out … so I think that leads us well into interdisciplinary work (R11. Medical anthropologist)

All participants reported having worked with researchers from other disciplines, mainly anthropology, epidemiology and public health. Some mentioned psychology, sociology, health policy and environmental sciences. One participant had worked with engineers.

Almost all participants described benefits of incorporating perspectives and insights from different disciplines. Working with different disciplines allowed some participant to learn new methods. However, incorporating different ways of working and looking at things was sometimes a challenge. Most described needing to ‘find a common vocabulary’ to communicate across disciplines. This was particularly important regarding context because of disciplinary differences in its conceptualization and study.

You have to learn, you have to socialize to each other’s languages.because you’re trained differently, with different models, you learn to find certain questions more important than others (R1. Medical anthropologist)

For example, one medical anthropologist reported that working with health psychologists was sometimes difficult because they focused on behavioral risk factors, such as smoking, whereas an anthropologist is more interested in ‘*why*’ someone smoked. Other participants said that medicine tends to prioritize individual-level factors, whereas anthropology is more concerned with the wider context. Personality and ego were also sometimes seen as a barrier for collaborations and interdisciplinarity.

### Popularity and relevance of syndemics

All participants agreed that syndemics is a growing field: it is receiving more attention from funders and from researchers with different backgrounds. However, participants pointed out that the concept is not always used appropriately or accurately, articulating all its components. For example, one participant reported having conducted a review on syndemic literature that found widespread misuse of the term. All participants agreed that it would be relevant to spread knowledge and use of syndemics, particularly outside anthropology.

Participants reflected on the relevance of syndemics for COVID-19. One participant was concerned about the risk of using the term prematurely, with no data or analysis available. Others argued against a general ‘COVID syndemic’; rather there may be situations for which the syndemic approach can be particularly relevant. In general, participants agreed that there are good reasons and opportunities to investigate syndemic interactions between COVID-19, other health conditions (e.g. obesity) and contextual factors, including marginalization or access to care.

## Discussion

Drawing on a literature review of syndemics research on NCDs and mental health and interviews with researchers in the field, this article examined how – context has been defined and studied. The analysis focused on how context is conceptualized, what study designs and methods are employed, and the possibilities for and challenges of interdisciplinarity in syndemics research.

Within the analyzed subgroup of syndemics, when studying context, emphasis was generally placed on factors that affect a specific population group and micro-level contextual factors (i.e. ‘living and working conditions’ and ‘social and community networks’). Calls for greater analysis of the structural factors that underpin the micro-level contextual factors [4,7] were echoed by some of the participants, who also recognized the challenges of doing so because of the complex relationship between macro socio-economic and policy process and local or individual drivers.

Focusing on micro-level context may also limit the scope of possible responses to the syndemic. Researchers have highlighted the potential of syndemics in terms of identifying options beyond medical interventions to prevent or mitigate health impacts [2,7,9]. Although highlighting the role of context has the potential to engender a more ‘socially conscious’ medicine and more integrated care [3], only by linking micro with macro social, economic, environmental processes can syndemics help to target the underpinnings of the health inequalities experienced by society’s most vulnerable [6,33,34].

Overall, the methodological gaps identified – a lack of longitudinal and multi-level studies – reflect those described by Tsai et al. [9]. Also, although respondents deemed a mixed-methods design most appropriate for syndemic research, the reviewed sources drew mostly on qualitative methods, particularly in-depth interviews. Such study designs help to identify and understand the circumstances where a syndemic emerges. Quantitative and population-level studies help to map to what extent population groups are affected and potentially allow the ‘measurement’ of interactions among syndemic components. Whereas longitudinal studies can more fully capture complex interconnections among syndemic components and help to clarify their relationships. The findings suggest that the field, especially the understudied NCD-related syndemics, could benefit from research designs with integrated quantitative and qualitative methods that would ideally use a longitudinal approach and recognize the importance of studying the connections between multiple levels of context. Recent qualitative research in Puerto Rico highlights the benefits of using a longitudinal approach in terms of describing the connections between context and interacting health conditions, but quantitative data collected alongside could have strengthened the argument [35].

Such an approach, however, brings clear challenges. Syndemic theory highlights how health and health issues are entangled in complex – biological and social – processes. Studying these biosocial phenomena [2] would hence benefit from expertise from diverse fields and researchers who are comfortable with communicating across disciplinary boundaries. As the participants indicated, epistemological debates and misunderstanding may arise when working across/with different disciplines. Context is one key term that is often understood differently among disciplines, such as psychology or anthropology. The findings, however, offer some reasons for optimism: participants’ professional experiences of interdisciplinary researchers reflected those described by Guimarães et al. [36] who described ‘*openness and tolerance*’ and willingness to learn from other disciplines. Bringing together insights and perspectives from different fields can foster the use of multiple data sources, helping to fulfil the methodological gaps within syndemic research.

Incorporating approaches from across disciplines into syndemic research is more crucial than ever because this may help to understand vulnerability to and the diverse impacts of the COVID-19 pandemic [37]. As pointed out by Irons [38], COVID-19 seems to disproportionally affect vulnerable population groups. Growing evidence indicates that COVID-19 morbidity and mortality rates are higher in people with pre-existing medical conditions, suggesting multiple biological interactions [39–41]. COVID-19 infection and health impact also seem to be linked to health and social inequalities [42]. Researchers have quickly recognized this: although at the time of the first interview no scientific articles had been published on COVID-19 and syndemics, a month later, there were over a hundred articles on this topic. For example, the Black community in the US was described as being in the center of a syndemic [43]. Also, Motta et al. [40], for example, highlighted on COVID-19 and TB prevalence in migrants, suggests other COVID-related syndemics.

### Strengths and limitations

The review focused on a specific group of syndemics, involving NCDs and mental health conditions. Research in this area has begun more recently than on HIV-related syndemics. This likely explains the small number of articles identified. The interviews provided more general information on how context is studied in other syndemics. Moreover, the findings from the review align well with those from the interviews.

Participants mostly described a background in anthropology. This limited the opportunity to identify disciplinary differences related to studying context. It also highlights how anthropology remains the leading syndemics discipline. Efforts to reach out to additional researchers from other disciplines were unsuccessful.

## Conclusion

The systematic review of research on NCD/mental-related syndemics and interviews with experts in the field revealed a relatively consistent working definition of syndemics. Context was broadly defined, with a tendency to focus on the micro-level. Methodological gaps, particularly lack of longitudinal and population-level analyses, were identified. Many respondents and study authors were anthropologists and they called for additional disciplines to participate in syndemic research. Nonetheless, respondents identified challenges for interdisciplinary within syndemic research, including different conceptualizations of context. Fostering and strengthening syndemic research can help understand how disadvantaged and marginalized populations experience disproportionated health impacts, which is relevant to the analysis of COVID-19-related morbidity and mortality.

## Paper context

Interactions between the social/economic context and disease conditions are a requisite of syndemics. However, context has varied definitions and elicits diverse study designs. Its use in syndemics research has seen little attention. This review of syndemics research and interviews with experts revealed a focus on micro-level context, a need for analysis of structural factors, and a lack of longitudinal or mixed-methods studies. Meanwhile, the unequal impacts of COVID-19 highlighted the need for interdisciplinary syndemics research.

## Appendix A. Literature review and participants information

**Table A1.** Summary of reviewed articles

Citation	Discipline^1^	Study design^2^	Main contextual factors analysed^3^
The syndemics of diabetes and depression in Brazil – An epidemiological analysis [21].	Social medicine	Quant	Living and working conditions Social and community networks Only partially addressed: General social, economic, cultural and environmental conditions
‘Just One Thing after Another’: Recursive Cascades and Chronic Conditions [22].	Anthropology	Qual	Living and working conditions Social and community networks Only partially addressed: Individual lifestyle factors
Association of Social Adversity with Comorbid Diabetes and Depression Symptoms in the Hispanic Community Health Study/Study of Latinos Sociocultural Ancillary Study: A Syndemic Framework [23].	Psychology	Quant	Living and working conditions Social and community networks
When HIV is ordinary and diabetes new: remaking suffering in a South African township [16].	Medical Anthropology & Public Health	Qual	Living and working conditions Social and community networks
Syndemic suffering in Soweto: Violence and inequality at the nexus of health transition in south Africa [15].	Medical Anthropology	Qual	Living and working conditions Social and community networks
Stress, diabetes, and infection: Syndemic suffering at an urban Kenyan hospital [24].	Medical Anthropology & Public health	MM	Living and working conditions Social and community networks
Addressing Syndemic Health Disparities Among Latin Immigrants Using Peer Support [25].	Anthropology	MM	Living and working conditions Social and community networks Individual lifestyle factors.
Transactions in Suffering: Mothers, Daughters, and Chronic Disease Comorbidities in New Delhi, India [26].	Medical Anthropology	Two case studies from a bigger MM study	Social and community networks
Applying Syndemics and Chronicity: Interpretations from Studies of Poverty, Depression, and Diabetes [27].	Medical Anthropology	Two case studies from two original MM studies	Living and working conditions Social and community networks Only partially addressed: Individual lifestyle factors.
The Utility of a Syndemic Framework in Understanding Chronic Disease Management Among HIV-Infected and Type 2 Diabetic Men Who Have Sex with Men [28].	Medicine & Public Health	Quant	Social and community networks Individual lifestyle factors Only partially addressed: Living and working conditions
When Diabetes Confronts HIV: Biological Sub-citizenship at a Public Hospital in Nairobi, Kenya [29].	Medical Anthropology	3 case studies narratives from a bigger MM study	General social, economic, cultural and environmental conditions Also included in the measure and analysis: Living and working conditions Social and community networks
Encountering Work: Intergenerational Informality, Child Labor, and Malnutrition in Urban Ecuador [30].	Anthropology	Qual	Living and working conditions Only partially addressed: General social, economic, cultural and environmental conditions.
Carolina in the Carolines: A Survey of Patterns and Meanings of Smoking on a Micronesian Island [31].	Anthropology	MM	Individual lifestyle factors Mainly, but embedded in a broader analysis including: Living and working conditions Social and community networks

^1^Discipline was determined by looking at the (main) professional title of first and second author(s).

^2^Quantitative (quant), qualitative (qual), mixed-methods (MM).

^3^Rainbow model layers [19].

**Table A2.** Participants information

Participant (P)	Professional background^1^	Type of syndemic-related research
1	Medical Anthropology	Research (fieldwork)
2	Medical Anthropology & Public Health	Research (fieldwork)
3	Medical Anthropology & Public Health	Research (fieldwork)
4	Medicine & Social medicine	(Quantitative) Research
5	Medical Anthropology	Academic debate and publications; research (fieldwork)
6	Public Health Anthropology	Academic work and debate (theorization of syndemic and other publications)
7	Medical Anthropology	Research (fieldwork)
8	Psychiatry	Academic debate and (quantitative) research
9	Public Health Nursing	Research (fieldwork) and intervention research
10	Epidemiology and Medical Anthropology	Research (fieldwork) and intervention research
11	Medical Anthropology	Academic work and debate (theorization of syndemic and other publications)

^1^Professional background was determined by looking at the (main) professional title of participants.
